# Supplementary material for: Global identification and mapping of socio-ecological production landscapes with the Satoyama Index
Source: PLoS One. 2021 Aug 18;16(8):e0256327. doi: 10.1371/journal.pone.0256327 (PMC8372939; doi:10.1371/journal.pone.0256327)
Supplement: Attachment — Submitted filename: Response to reviewers.docx [file pone.0256327.s003.docx]

**RE: Response to reviewers of our original submission**

Dear PLoS One eidtors,

We have addressed the weaknesses pointed out about the original submission by the reviewers, which led us to substantial revise the texts and redo the analysis using the new datasets. The major revisions are:

Reconsideration of the way we set the threshold for SEPLS along the Satoyama Index spectrum. We now use a statistical analysis to establish the thresholds regionally. In doing this, we created two new datasets for this analysis. We believe our analysis is more robust now.

The discussion about the behavior of the Satoyama Index in the Amazon deforestation areas was rather weak and not grounded on data. In this revised manuscript, we present the explanations of the problematic behavior there using the interpretation of the land use/cover data that we used (GLCNMO2013, with comparison to GLCNMO2008).

The discussion has been revised according to the new findings from the new analysis. Thus, we would like our manuscript re-considered for PLoS One. Below, we provide the point-by-point response to the comments made by the two reviewers on our first manuscript in the bold font.

Best regards,
Yoji Natori
Corresponding Author

**-----**

**Comments to the Author**

1. Is the manuscript technically sound, and do the data support the conclusions?

The manuscript must describe a technically sound piece of scientific research with data that supports the conclusions. Experiments must have been conducted rigorously, with appropriate controls, replication, and sample sizes. The conclusions must be drawn appropriately based on the data presented.

Reviewer #1: No

Reviewer #2: Yes

**With the reanalysis and revisions, we believe that the concerns of Reviewer #1 have been addressed.**

2. Has the statistical analysis been performed appropriately and rigorously?

Reviewer #1: N/A

Reviewer #2: Yes

**Our original analysis did not use a statistical analysis, but the current manuscript does.**

3. Have the authors made all data underlying the findings in their manuscript fully available?

The [PLOS Data policy](http://www.plosone.org/static/policies.action#sharing) requires authors to make all data underlying the findings described in their manuscript fully available without restriction, with rare exception (please refer to the Data Availability Statement in the manuscript PDF file). The data should be provided as part of the manuscript or its supporting information, or deposited to a public repository. For example, in addition to summary statistics, the data points behind means, medians and variance measures should be available. If there are restrictions on publicly sharing data—e.g. participant privacy or use of data from a third party—those must be specified.

Reviewer #1: No

Reviewer #2: Yes

**We did make the data underlying our findings available in the manuscript and in the supporting document with out original submission. We are doing the same with the submission this time. We understood that the assessment of Reviewer #1 here came from the assessment provided below. If else, we would appreciate it very much if we are informed of the specifics.**

4. Is the manuscript presented in an intelligible fashion and written in standard English?

PLOS ONE does not copyedit accepted manuscripts, so the language in submitted articles must be clear, correct, and unambiguous. Any typographical or grammatical errors should be corrected at revision, so please note any specific errors here.

Reviewer #1: Yes

Reviewer #2: Yes

**Thank you. We maintain the same level of care in this submission.**

5. Review Comments to the Author

Please use the space provided to explain your answers to the questions above. You may also include additional comments for the author, including concerns about dual publication, research ethics, or publication ethics. (Please upload your review as an attachment if it exceeds 20,000 characters)

**Reviewer #1:** This paper evaluated global landscapes using the existing indicator, i.e. Satoyama Index (SI). Data sources and computation procedures of SI were well described and calculation results seemed to be reasonable in light of the previous literature (i.e. Kadoya and Washitani, 2011). However, I have several serious concerns and some minor comments as below.

Major concerns
1. The major purpose of this paper is to see whether SI can be a good indicator to evaluate the global distribution of SEPLS. Essentially, landscape diversity (or mosaic structure) is only one aspect of SEPLS, which can be more broadly characterized and conceptualized by many other factors, e.g. farming practices, local knowledge and cultural relations. SI lacks these perspectives and thus could possibly show higher values in the areas where deforestation and subsequent land development are ongoing, as indicated by the authors. They argued that it “is showing the areas where sustainable land use practices must be put in place”, but if so, this indicator does not necessarily show the current distribution of SEPLS. Also, they presented the SI values of SEPLS-related project sites to claim the appropriateness of this indicator, however, higher values in these sites seem somewhat natural because they would have been chosen due to their suitability for the SEPLS definition including landscape diversity. Yet, lower values were also found in some project sites. If higher values could be shown in non-SEPLS sites (e.g. deforesting areas) and lower values could be attached in even SEPLS-related project sites, how can we use this indicator to evaluate SEPLS distribution? Briefly, the appropriateness of SI to evaluate SELPS may not be justifiable at least with the current presentation.

**Thank you very much for pointing this out. We considered this point seriously and undertook reanalysis using new datasets that enabled a statistical analysis. The analysis is not the extension of the one in the original submission, but a completely new analysis. We believe that the new analysis removes the concerns raised on our original submission.**

2. This paper critically lacks literature reviews on the relevant topics. For examples, the methods to evaluate cultural landscapes, which is the socio-ecological concept similar to SEPLS, have been proposed by some scholars (e.g. Arnaiz-Schmitz et al., 2018; Martínez et al., 2010; Tieskens et al., 2017), but these are not mentioned in this paper. What are the advantages of SI over other indicators that integrate additional perspectives (e.g. management intensity, social welfare and spatial fragmentation) into landscape evaluation? Without literature reviews, uniqueness and importance of the paper will remain obscure.

**Thank you very much for the suggestion of the literature. We found Tiesken et al. (2017) very relevant to our study, and thus included in this submission. The other two seemed less so, and thus we decided not to include. We significantly enhanced our literature review on the aspects in line with our new discussion.**

3. One of the major conclusions of this paper came from the subsection “Areas of biodiversity significance” under “Discussion”. Nevertheless, this topic was not mentioned in the introduction and others, and thus this conclusion seemed to appear out of nowhere. Also, the authors claimed that 35% of lands, which is covered by either protected areas, KBA or SEPLS (higher SI sites more precisely), should be under sustainable practices. Two issues merit comments. One is that the authors should clarify the meaning of sustainable practices in protected areas, some of which restrict human interventions (e.g. wilderness area). The other is that calculation method is not provided. Supposing that the number of land units that fall within the above three categories is 1,820,241 (my own calculation), and this is equivalent to 35.4% of lands excluding Antarctica, total number of land units considered in this analysis will be 5,141,923. Given that the total land units are 8,571,076 as indicated in the paper, Antarctica would dominate around 40% of global terrestrial areas, which is much higher than the actual value (i.e. around 9%). Using this actual value results in 7,799,679 total land units and thereby decrease the share of land units under three categories to 23.3%. I may have misunderstood some computation procedures, but the authors must indicate their methodologies to do this, because this is one of the major conclusions of this paper.

**The areas of biodiversity significance (protected areas and KBAs) are relevant to the issues we present in the Introduction. We included the description of them in the Discussion section in the original submission, which has been moved to Methods section per comment from Reviewer #2. We hope this addressed the concern of Reviewer #1 on this regard.**

**We stated in the original submission that the %s were computed using the number of land units, which admittingly differ from real physical area (especially in the higher latitudes, such as the Antarctica). On this point, too, we hope the relocation described above clarifies the confusions.**

4. The authors argued the necessity of longitudinal data to identify environmental degradation and thereby improve the index accordingly. This will be feasible, from my perspective, since time-series global land cover data is available at either GLCNMO or MODIS. The authors may be able to propose the improved index and compare its usefulness to the existing one in identifying SEPLS sites.

**We also hoped even in the preparation of the first submission that GLCNMO datasets served the basis for longitudinal comparison. We did not describe the reason we did not use GLCNMO at different times, but now we do. We provided the critiqued of the temporal consistency of the classification of GLCNMO as the explanation that made us unable to compare between 2008 and 2013 datasets. This also enhanced our discussion about the problematic behavior of the Satoyama Index in the Amason.**

**As a way to improve the index, our argument has changed based on the new findings that we present now. We argue that the Satoyama Index is valid at the global level (which matters the most for us) and that details at finer scales (thematic resolution, etc.) should be addressed in the areas of interest according to the local circumstances.**

Minor comments
1. L62-64: It will be preferable to cite any supporting literatures.
2. L74: Where do we have the current global map of SEPLS?
3. L90-91: The reason why the authors did not use 30-sec resolutions should be provided.
4. L138-141: The resolution is not high enough to see this.
5. L212-214: This sentence may not be quite convincing to the readers. The reason why the value of 0.5 (instead of the mean 0.43 or the median 0.48) is used needs to be more elaborated.
6. L364: This long table should be moved to Appendix, considering its smaller contribution to the overall conclusion of this paper.
7. L39-382: How can you identify the potential trade-offs with the SI map? More elaborations will be preferable.

**All the minor points raised have either been addressed or became irrelevant due to the reanalysis. We are providing maps in higher resolutions now; if the resolution is low in the PDF, please refer to the original TIF image.**

**Reviewer #2:** This manuscript reported that validity and limitation of Satoyama Index (Kadoya & Washitani 2011), an indicator of traditional agricultural landscapes with high biodiversity, through aggregating the index values within areas supported by international programs for achieving societies in harmony with nature (or protected areas). This study is carefully summarized the results and will contribute to decision-making on production landscapes which plays important role in biodiversity conservation. However, some correction and addition of information should be done to make the manuscript more suitable for publication. Please see comments below.

L49. Please insert a brief sentence explaining the essence of Satoyama Index after the “SEPLS globally” For example, “The Satoyama index is based on diversity of land classes within an agricultural landscape and obtained from landcover/landuse maps...”

L51. Please remove “)” after “SEPLS”.

L75. Please explicitly show what you examined or mentioned hypothesis in this part, before “discuss how it can used...”

**The above points by Reviewer #2 have been addressed.**

L116-119. From what point of view, these sites were selected as project sites of these project and why authors picked up in the study? It should be explicitly described here or the last paragraph of introduction for readers who are not familiar with a Japanese word “satoyama”. I think that these sites can be representative SEPLS which can play important role in promoting societies in harmony with nature and selected from comprehensive and multipurpose perspective (maybe from complex, qualitative, and political reasons, and at least, selected independently from the Satoyama index), while Satoyama index is based on a snapshot of landcover/landuse and targeting biodiversity (which may be relatively quantitative and objective, but has weakness which authors pointed in the manuscript). However, it is not explicitly shown.

**We now use different sets of sites in our analysis, and we describe these datasets in the Methods section. We hope this clears this concern.**

L301-312; L339-345. Please move this part to the Materials and Methods.

**Thank you for the suggestion. We did.**

L318-321; L345-350, Table 2. Please move this part to the Result.

**This table and associated section has been removed in the current submission.**

L401. About the problem on the thematic resolution and the number of land use/cover, a new calculation method for the satoyama index which can downweight “ecologically redundant” land class was proposed by Yoshioka et al. (2017). Please mention that the well-customized Satoyama index using the method may be effective at the regional level.

**The context of this issue has changed from our first submission, but this suggestion has been incorporated in our current submission.**
