# Supplementary material for: Global identification and mapping of socio-ecological production landscapes with the Satoyama Index
Source: PLoS One. 2021 Aug 18;16(8):e0256327. doi: 10.1371/journal.pone.0256327 (PMC8372939; doi:10.1371/journal.pone.0256327)
Supplement: Attachment — Submitted filename: Responses to reviewers.docx [file pone.0256327.s004.docx]

**RESPONSE TO THE REVIEWERS**

We respond to the comments raised by the reviewers as follows. Descriptions of our actions follow the comments made (indicated by ** and in bold font).

--------

Journal Requirements:

When submitting your revision, we need you to address these additional requirements.

1. Please ensure that your manuscript meets PLOS ONE's style requirements, including those for file naming. The PLOS ONE style templates can be found at

<https://journals.plos.org/plosone/s/file?id=wjVg/PLOSOne_formatting_sample_main_body.pdf> and

https://journals.plos.org/plosone/s/file?id=ba62/PLOSOne_formatting_sample_title_authors_affiliations.pdf

****We followed the PLOS ONE style requirements.**

2. Please ensure that you include a title page within your main document. We do appreciate that you have a title page document uploaded as a separate file, however, as per our author guidelines (<http://journals.plos.org/plosone/s/submission-guidelines#loc-title-page>) we do require this to be part of the manuscript file itself and not uploaded separately.

Could you therefore please include the title page into the beginning of your manuscript file itself, listing all authors and affiliations.

****The title page is now included as the first page of the “manuscript.”**

3. Thank you for stating in your Funding Statement:

'This study was funded in part by a grant from the Global Environment Facility to Conservation International Japan for the “GEF-Satoyama Project” (Project ID: 5784; <http://www.thegef.org/projects>). The funders had no role in study design, data collection and analysis, decision to publish, or preparation of the manuscript.'

a. Please provide an amended statement that declares *all* the funding or sources of support (whether external or internal to your organization) received during this study, as detailed online in our guide for authors at <http://journals.plos.org/plosone/s/submit-now>

Please also include the statement “There was no additional external funding received for this study.” in your updated Funding Statement.

b. Please include your amended Funding Statement within your cover letter. We will change the online submission form on your behalf.

****Revised Funding Statement was included in the cover letter.**

4. We note that Figures 1, 3, 4 and 6 in your submission contain map images which may be copyrighted.

****The map images in question were provided by Natural Earth, which is in the public domain and the provider states that “crediting the authors is unnecessary.” However, in consultation with the Editorial Office, we have included the following statement at the end of the captions of the figures concerned: *(Made with Natural Earth; free map data at naturalearthdata.com)*.**

5. Please ensure that you refer to Figure 5 in your text as, if accepted, production will need this reference to link the reader to the figure.

****The cross-referencing in WORD had error during the PDF conversion. It has been corrected.**

Additional Editor Comments:

Thank you for your manuscript. We acquired reviews from four reviewers and they vary widely, ranging from a recommendation to reject (Reviewer 2) to major and minor revision recommendations (Reviewers 1 and 3) and even an "accept with editing for English usage" (Reviewer 4). After reading through the reviews and also the manuscript, I believe some somewhat substantial revisions are necessary to improve the manuscript and make it appropriate for publication. Specifically, please address the comments of Reviewer 1, Reviewer 3, and Reviewer 4, which are either fairly detailed (as in recommending specific edits and changes) or a bit general (improve English usage). Each of these reviewers indicates some clear changes, but also asks some specific questions. Please be sure to make changes which respond to these.

Although I did not chose to fully accept reviewer 2's recommendation to reject this manuscript at this time, I do believe you must add a section addressing how the Satoyama Index can be extended to apply to more broadly defined landscapes, or why Reviewer 2's assessment has shortcomings. In this section, please address a broad audience, not just Reviewer 2, but be sure to do justice to their statements.****The purpose of the study was to demonstrate the global applicability of the Satoyama Index. We edited our manuscript to emphasize this point. We also changed the beginning of the Discussion section to discuss this point.**

Finally, I agree with Reviewer 1 that the data should be made available in an open format if possible, but would note that a File Geodatabase (*.gdb) is accessible in open source software through the GDAL framework (<https://gdal.org/drivers/vector/openfilegdb.html>)
****Layers in the File Geodatabase file are now provided in the Shapefile format.**

**Comments to the Author**

1. Is the manuscript technically sound, and do the data support the conclusions?

The manuscript must describe a technically sound piece of scientific research with data that supports the conclusions. Experiments must have been conducted rigorously, with appropriate controls, replication, and sample sizes. The conclusions must be drawn appropriately based on the data presented.

Reviewer #1: Yes

Reviewer #2: No ****A discussion on broader applicability of the Satoyama Index has been added.**

Reviewer #3: Yes

Reviewer #4: Yes

2. Has the statistical analysis been performed appropriately and rigorously?

Reviewer #1: Yes

Reviewer #2: Yes

Reviewer #3: Yes

Reviewer #4: Yes

3. Have the authors made all data underlying the findings in their manuscript fully available?

The [PLOS Data policy](http://www.plosone.org/static/policies.action#sharing) requires authors to make all data underlying the findings described in their manuscript fully available without restriction, with rare exception (please refer to the Data Availability Statement in the manuscript PDF file). The data should be provided as part of the manuscript or its supporting information, or deposited to a public repository. For example, in addition to summary statistics, the data points behind means, medians and variance measures should be available. If there are restrictions on publicly sharing data—e.g. participant privacy or use of data from a third party—those must be specified.

Reviewer #1: No ****GIS data layers are now provided in the Shapefile format.**

Reviewer #2: Yes

Reviewer #3: Yes

Reviewer #4: Yes

4. Is the manuscript presented in an intelligible fashion and written in standard English?

PLOS ONE does not copyedit accepted manuscripts, so the language in submitted articles must be clear, correct, and unambiguous. Any typographical or grammatical errors should be corrected at revision, so please note any specific errors here.

Reviewer #1: Yes

Reviewer #2: Yes

Reviewer #3: Yes

Reviewer #4: No ****We have received English proofreading.**

5. Review Comments to the Author

Please use the space provided to explain your answers to the questions above. You may also include additional comments for the author, including concerns about dual publication, research ethics, or publication ethics. (Please upload your review as an attachment if it exceeds 20,000 characters)

Reviewer #1: This manuscript reported that socio-ecological production landscapes (SEPLS) can be mapped using the Satoyama Index (Kadoya & Washitani 2011), an indicator of traditional agricultural landscapes with high biodiversity. Following the reviewer’s comments, the authors substantially revised the manuscript; they vigorously collected data on spatial distribution of SEPLs (terrestrial socio-ecological production landscapes) and conducted statistical test to compare the values of Satoyama index inside and outside the SEPLs. Although the manuscript was greatly improved, some modifications will make it more suitable for publication. Please see comments below.

L51-79. Rearrangement of the paragraphs will make the part easier to read. For example, the part on “SEPLS and area-based conservation prioritization” (L51-59) can be moved to immediately after the part on OECM (L70-79). Then, the sentences in L66-69 can be moved to head of the paragraph on importance of geographic information of SEPLS(L80-84). Please note that some corrections of sentences should follow the rearrangement (For example, “At the same time, it…” should be corrected to “However, IPSI database presented a weakness that…” if the sentences moved to the head of the L80.) ****Thank you very much for the suggestion. The manuscript has been revised to reflect these suggestions.**

L64-66. Although the authors assumed that there are potential SEPLs which have not registered in the IPSI database (and will be found by Satoyama index) in the world, it was not explicitly mentioned here. The large number of the cases in the IPSI database can give readers the impression that the database covers the SEPLs in the world. So, I recommend that authors explicitly mention the assumption here. ****Thank you for pointing out an important point. We addressed this as part of our edits in the previous para.**

L189. Please clarify the spatial scale of a 3*3 clusters (6km * 6km?). I think that the authors meant 3*3 cells (or pixels) here, it should be difficult to understand for readers unfamiliar with GIS data. ****Clarified that this refers to the clusters of land units, i.e., nine 6km grid cells.**

L190. Please correct “he” to “the”. ****Done.**

L193-194. Is the dataset of “9-neighbors” included in the Supplement material? If so, please cite it (e.g. data S1 etc.). ****S1 File contains all GIS layers that we produced and used in this paper. We added a sentence to indicate this fact in the section for Satoyama Index validation, where the content of the S1 File is first mentioned.**

L211. Was the assumption of normality of the data checked? (Although I do not think it critically affect the result)
In addition, I recommend the authors to attach that the raw data used in the statistical analysis in the form of an Excel file (or csv or txt) as a supplement.

****We found the data distributions were not normal by Shapiro-Wilk normality test, which led us to retry the statistical analyses with nonparametric means. The Wilcoxon rank sum tests revealed the same results as we reported initially with the t-test. We replaced Table 2 and revised the texts accordingly.**

L279. Fig. 7 should be cited here. ****As we do not want to refer to a figure out of sequence, we chose to mention the section heading where relevant information is presented.**

L305. Citation error. Fig. 5? ****Yes. Corrected.**

L319-361. The problem on Amazon does not appear to be improved by the point mentioned in the “points for improvement”. Considering that newer land classification had made the problem more serious, simple improvement of satellites or base landuse/landcover maps may not be effective. So, please add the idea (or hypothesis) which may systematically resolve the problem to the end of the section or the part on the points for improvement. For example, consideration of the history of farming may be a key. The result of meta-analysis by Queiroz et al. (2014) suggested that the contribution of land abandonment to biodiversity differed between the old and new continents. In addition, consideration of spatial unit of unsustainable crop rotation may also be important. In the Amazon, newer GLCNMO may correctly classified wild (abandoned?) open habitats owning to crop rotation, which is usually unsustainable and less important for biodiversity in the region. The problem of overvaluation of non-crop habitats following crop rotation may have been overlooked in the region where rice paddies are dominant, because rice paddies do not need crop rotation from the view point of sustainability. So, there is a room for improvement in the Satoyama index, which originally focused agricultural landscapes including rice paddies.
****We added a paragraph at the top of the section to discuss this point.**

L670. The “gdb” is a special file format for ArcGIS. The file should be shared in the form which can be opened by the other free software such as QGIS. ****The GIS data are now provided in the Shapefile format.**

Reviewer #2: The manuscript presents a very simple index developed for specific agricultural and sustainable-use landscapes in Japan that are often protected. The index can work very well in such landscapes where the heterogeneity of the landscape associated with the cultural model is constant. But I don't see that it can work in other types of landscapes with different degrees of heterogeneity associated with different cultural models. In fact the authors find abnormally high and low values in different circumstances. The applicability of this simple index, which does not consider the social economic model, nor the associated ecological functioning of landscapes, seems to me to be impossible for the detection of productive socio-ecological landscapes in places other than the productive landscapes of Japan. It can work to detect differences between the productive landscapes of Japan, but the idea of its global applicability does not seem possible to me. However, the manuscript is well written and statistically adequate. I would recommend the authors to adapt the index for use in a globalized framework.

****It is true that Satoyama Index was developed by scholars in Japan and validation was done primarily with data from Japan. The critiques are valid, and they form precisely the basis of our study; can Satoyama Index be used to identify SEPLs outside Japan. Our validation data came from other counties in different socio-economic circumstances, different forms of agriculture, and different climatic conditions. They were selected independently from the values of the Index, and our investigation, in effect, as to determine whether statistically significant correlation existed.**

Reviewer #3: Major comments:
The paper outlines the use of the Satoyama Index and its usage and potential for mapping and understanding diverse production landscapes. This reviewer is not an expert in statistical analysis, but it appears to make a good case for its argument, based on the research and analysis provided. One point is that it could well use a native proofreader to look through it as there are many minor issues with the language. ****The revised manuscript received a native proofreading.**

Minor comments (I will not note the many minor errors in English that could be addressed by a native check but rather focus on substance and only places that are likely to be missed by a proofreader if they are unfamiliar with the content):
3: “Satoyama” is not a one-to-one term for the term used here “socio-ecological production landscapes”, but one example of such landscapes in Japan. Would recommend something like “Satoyama, a kind of socio-ecological…”.

****Thank you for pointing this out. There is a wide spectrum of what is considered/called as “Satoyama,” even in the IPSI fora, which is precisely why a clear definition is important. We admit that the use of terms, Satoyama and SEPLS/SEPLs was not consistent. We addressed this issue in the revised manuscript.**

42: My reading is that CBD Decision X/32 only “recognizes” and “takes note of” the Satoyama Initiative, but does not “adopt” it. ****The word “adopt” was used casually, but it was not accurate when it referred to an international convention. We rephrased it to address this point.**

59: The statement that “In this regard, SEPLs can be considered as a conservation priority” needs to be qualified, as it does not seem that it is currently considered a conservation priority. Something like “SEPLs seem like a good candidate to be a conservation priority” or the like could be better. ****Thank you. The candidacy capture the sense we wanted to mean perfectly. Revised accordingly.**

62: I think the word “natural” should be “national”. ****Very true. Thank you.**

183: “IPSI” is misspelled here as “ISPI”. An overall check should be done in case this error occurs elsewhere. ****Corrected and checked throughout the manuscript.**

451-455: The concept of OECMs is raised here, and then immediately negated in the sentence “Whether as OECM or not…”, making it unclear how OECM is relevant to the argument. ****We revised our argument for clarity.**

455-456: The percentage of land that should be covered in this kind of global biodiversity policy is a very salient issue in CBD negotiations, and defending the need for 20% seems like it would require much more attention than this casual mention here. It seems beyond the scope of the current paper, unless the authors would like to greatly expand on how the figure of 20% is reached. ****We decided to remove the discussion of 20% for appropriate management. Instead, we linked the rest of the information in this paragraph to the discussion of OECM.**

481-482: It would be helpful to at least give the headline topics of the SDGs listed here, “Zero Hunger” and “Live on Land”. ****Done.**

484-485: “people-nature coexistence” – the term “nature-social interactions” is used in the abstract. Is this terminology that is or should be made consistent? As a reader I am not sure if it is exactly the same thing. In the same vein, the term “landscapes of people-nature coexistence” is new here. Is it the same as SEPLs? ****We rephrased the relevant parts to avoid causing confusions.**

487-492: This is mentioned earlier in the paper as well and it may be beyond the scope of the current paper, but this seems like the major issue. It is unclear how the essential problem of having data at a sufficient resolution will ever be possible particularly for huge areas of land, and therefore if this kind of research can ever produce the results desired. If there is any more information on the potential of how this can be done practically it would be helpful for the paper. ****Our point here is to remind that the scale of global assessment is not sufficiently applicable for more locally-focused interest, and that more detailed studies might be conducted in regions that have been narrowed by the global Satoyama Index assessment. We edited the relevant parts to clarify this point.**

Reviewer #4: The article is significant in the conservation of SEPLs and can be a useful tool for policy makers. Well researched and supported by data. However, the manuscript should be edited and should be written in standard English since some of the sentences are in first person (e.g. L103, L467). The abstract should also be edited since there are missing information. The abstract should stand alone and when read should provide the summary of the research. Overall, the paper is acceptable.

****We do not agree that the use of first-person voice is not the standard English, so we did not change those sentences. However, we received a native proofreading to address other issues regarding English grammar and to improve clarity. We revised the abstract as suggested.**
